# Informational efficiency and welfare

**DOI:** 10.1007/s11579-022-00319-3

**Published:** 2022-05-26

**Authors:** Luca Bernardinelli, Paolo Guasoni, Eberhard Mayerhofer

**Affiliations:** 1 London, UK; 2 School of Mathematical Sciences, Dublin City University, D09 W6Y4 Dublin, Ireland; 3 Dipartimento di Statistica, Università di Bologna, Via Belle Arti 41, 40126 Bologna, Italy; 4 Department of Mathematics and Statistics, University of Limerick, V94 T9PX Limerick, Ireland

**Keywords:** Equilibrium, Rational expectations, Heterogeneous information, Welfare, G11, G12, G15, 91G10, 91G80

## Abstract

In a continuous-time market with a safe rate and a risky asset that pays a dividend stream depending on a latent state of the economy, several agents make consumption and investment decisions based on public information–prices and dividends–and private signals. If each investor has constant absolute risk aversion, equilibrium prices do not reveal all the private signals, but lead to the same estimate of the state of the economy that one would hypothetically obtain from the knowledge of all private signals. Accurate information leads to low volatility, ostensibly improving market efficiency, but also reduces each agent’s consumption through a decrease in the price of risk. Thus, informational efficiency is reached at the expense of agents’ welfare.

## Introduction

Hayek [15] famously observed that *“knowledge never exists in concentrated or integrated form, but solely as the dispersed bits of incomplete and frequently contradictory knowledge which all the separate individuals possess”*. Since then, understanding the ability of markets to aggregate information dispersed among participants has been central to evaluate their efficiency. Indeed, each version of Fama’s market efficiency hypotheses [11] is a statement on the type of information that asset prices reveal.

The natural counterpart of informational efficiency is its impact on welfare, that is, the effect that information quality has on market participants. Such an effect is twofold: On one hand, each individual brings personal information to bear on market prices through demand for assets. On the other hand, the same individual benefits from the information latent in prices, which partially reveal the demand of other individuals. In contrast to informational efficiency, the impact of information aggregation on agents’ welfare has received considerably less attention in the literature.

This paper tackles these twin questions in a continuous time model, where agents differ in their constant absolute risk aversion, time preference, and, most importantly, private information. Each agent’s information provides a noisy signal on the state of the economy, which in turn affects the stream of dividends paid by a risky asset, in unit supply, whose price is determined in equilibrium. In making their consumption and investment decisions, agents have access to a safe asset, available in unlimited supply with constant interest rate. Crucially, each investor bids the risky asset based on the private signal and on the public information embedded in dividends, which are exogenous, and in prices, determined endogenously.

We find explicitly the linear equilibrium price, its implied *consensus* estimate of the state of the economy, and the resulting welfare for each agent, thereby obtaining a framework for answering the twin questions of informational efficiency and its welfare impact. The equilibrium displays two ostensibly contradictory results: (i) the market optimally aggregates agents’ private signals in prices, but (ii) such information reduces the welfare of each agent. Put differently, the intellectual gain from superior information is accompanied by an economic loss.

The first finding is consistent with the results on fully-revealing equilibria, pioneered in one-period models by Grossman [13], and generalized in [1, 10, 14, 16] to partial revelation by including noise traders or, equivalently, a noisy supply of risky assets, both of which make market information inaccurate.^1^ As our focus is on information aggregation, we eschew noise traders to concentrate on rational agents with separate private information flows. In a continuous-time model where agents maximize utility from consumption over an infinite horizon, we show that full revelation holds, though the market does not reveal all individual signals, but only a sufficient statistic for the state of the economy. Thus, in the same spirit as [22], we find that market prices transmit only relevant knowledge to participants, discarding redundant information. Our dividend dynamics is based on the asymmetric information framework of Wang [23], but we do not prescribe informed and noise traders, positing instead several agents, all rational and endowed with different information.

The difference between one-period and the present dynamic setting is more than technical. With a single period, current signals and prices are the only information available to agents, while in a dynamic setting present signals affect consumption and investment decisions even in the future, as rational agents take all past information into account to form their views of the state of the economy.

The second finding, on welfare, does not seem to have been investigated in depth in the literature, and the contrast between equilibrium informational gains and welfare losses deserves a closer examination of the mechanisms leading to both effects. A key aspect of this phenomenon is equilibrium volatility: intuitively, more information reduces the volatility of asset prices because more accurate predictions on future dividends decrease the standard deviation of unexpected shocks. The less intuitive implication of lower volatility is that the reduction in risk leads to a much lower risk premium available to investors, hence a deterioration of investment opportunities, which ultimately reduces consumption and welfare.

The paradox of informational gains with welfare losses rests on a number of assumptions, including constant absolute risk aversion of agents, normally distributed shocks, initial endowments in cash only, and the presence of a safe asset with constant rate, in unlimited supply (equivalently, a riskless technology with linear returns). Although these assumptions are ubiquitous in the literature and, in the absence of a general theory, the attribution of any conclusion is necessarily tentative, it is worth noting that the unlimited supply of safe assets is intuitively a key driver of the paradox, and helps qualifying its relevance.

In the model, the safe asset plays the fundamental role of store of value and is impervious to the flow of information. Thus, more information merely affects the valuation of the risky asset in relation to the safe one, without altering agents’ inclination to consume over time. As an inelastic safe rate allows agents to consume or save in the aggregate, more impatient agents have no reason to alter their risky holdings, as their fluctuations are inconsequential for short-term consumption decisions.

Furthermore, the presence of a safe asset in unlimited supply, in contrast to the assumption of zero supply typical of another strand of literature on general equilibrium, implies that the risky asset is not the only type of wealth in the economy, but merely a extra source of revenue, to be evaluated for its marginal contribution to long-term consumption.

The last observation suggests that the main result is relevant to understand the effect of competitive markets in aggregating the information on an individual asset from market participants, and in evaluating the effect of such aggregation on the residual value available to investors. In this context, the main message of the main result is that the lower volatility resulting from more information does not benefit investors, but rather pre-existing owners of the asset (such as a company’s founders and venture capitalists in an IPO), who see prices increase as a result of the lower risk premium. Vice versa, the assumption of an unlimited supply of safe asset does not endorse the same conclusions at the macro level, because in this context a substantial increase in information may also translate into a change in the equilibrium interest rate, both through variations in conditional dividend growth and through the precautionary-savings channel.

This paper also contributes to the literature on information aggregation by offering a rigorous treatment of admissible strategies, linear equilibria, and optimality in the presence of heterogeneous information. In the familiar setting of portfolio choice, prices and information flows are exogenous, while consumption and investment strategies are endogenous. In the other familiar setting of representative-agent equilibria, cash flows and information flows are exogenous, while prices are endogenous. In the present setting, cash flows are exogenous, but both prices and information flows are endogenous. Because each agent can choose from a different set of consumption and investment strategies, admissible strategies are agent-specific, and the filtration generated by market prices is also endogenous.

Finally, note that the paradox highlighted in this paper – that more information may lead to lower welfare – is reminiscent of but conceptually different from the the Grossman-Stiglitz [14] paradox on the impossibility of informational efficiency. In contrast to their model, in which one-period information acquisition is costly and noise traders are present, here each agent is endowed with a costless personal signal that flows continuously and can affect consumption and investment decisions, while noise traders are absent.

At the technical level, our model is closest to [20], with some important differences. While both models entertain infinite-lived agents with constant absolute risk aversion and individual signals, [20] focuses on a continuum of agents, so that the impact on aggregate demand of individual noises is null by design. On the contrary, we consider a finite number of agents, leaving aggregate noise to be random. A finite number of agents also allows us to find explicitly equilibrium quantities in terms of exogenous parameters, and does not involve the measurability issues arising with the aggregation of a continuum of independent random processes. In addition, [20] introduces noise traders by considering a fluctuating supply of shares, while we focus on a constant supply, thereby excluding noise traders.

The rest of the paper is organized as follows: Sect. 1 describes the model in detail and states the main result, characterizing the equilibrium asset price, its implied consensus estimate, and the optimal consumption-investment strategies of the agents. Section 2 discusses the main implications for information and welfare, separating the effects of dividend risk, state-of-the-economy risk, and heterogeneous information. Section 3 derives the equilibrium from control arguments. Concluding remarks are in Sect. 4. All proofs are in the appendix.

## Model and main result

### The economy

The economy includes a safe asset, in unlimited supply and paying a constant interest rate *r*, and a risky asset, in unit supply and paying the dividend stream $(D_t)_{t\ge 0}$ described by

1.1 $$\begin{aligned} dD_t=(\pi _t-k D_t)dt+\sigma _D dW^D_t, \quad D_0\in {\mathbb {R}}, \end{aligned}$$

where $k,\sigma _D>0$, $W^D$ is a Brownian motion and the state of the economy $(\pi _t)_{t \ge 0}$ is an Ornstein-Uhlenbeck process

1.2 $$\begin{aligned} d\pi _t=a({\bar{\pi }}-\pi _t)dt+\sigma _\pi dW^\pi _t, \end{aligned}$$

driven by an independent Brownian motion $W^\pi$, where $a, \sigma _\pi , {\bar{\pi }}>0$. In other words, the dividend stream $D_t$ grows at some time-varying rate $\pi _t-k D_t$ that depends on the current state $\pi _t$, which stochastically reverts to some long-term mean ${\bar{\pi }}$. Note that, although dividend shocks and state shocks are independent, $D_t$ and $\pi _t$ are dependent because the past values of the state $\pi$ affect the current dividend $D_t$.^2^ Furthermore, the initial value $\pi _0$ is assumed to be independent of $W^D, W^\pi$ and normally distributed with mean ${\bar{\pi }}$ and variance ${\overline{\sigma }}^2_\pi$.^3^

### Agents: objectives and information

There are *n* agents who invest in the safe and risky assets to maximize expected utility from consumption. Each agent *i* ($1\le i\le n$) has additive preferences with individual discount rate $\beta _i>0$ and constant absolute risk aversion $\alpha _i>0$.

None of the agents can see the underlying state of the economy $\pi _t$, but they all have access to public information, comprised of the dividend stream $(D_s)_{s\le t}$ and the price of the risky asset $(P_s)_{s\le t}$, to be determined in equilibrium, as discussed below. In addition, each of them sees a private signal $\xi ^i_t$, which offers a noisy glimpse of the latent state of the economy. Thus, for any $1\le i\le n$,

1.4 $$\begin{aligned} d\xi ^i_t= \pi _t dt+ \sigma _i dW^i_t, \qquad \xi ^i_0=0, \end{aligned}$$

where $(W^i)^{1\le i\le n}$ is an *n*-dimensional Brownian motion independent of $(W^D,W^\pi )$ and $\sigma _i>0$ for $1\le i\le n$. Note that the shocks to the signals of different agents are independent, though signals themselves are interdependent, as they are all affected by the state of the economy.

The probability space is $(\Omega ,{\mathcal {G}},({\mathcal {G}}_t)_{t \ge 0},{\mathbb {P}})$ where ${\mathcal {G}}_t$ is the augmented natural filtration of $\pi _0$, $W^D_u$, $W^\pi _u$, and $W^i_u$ for $1\le i\le n$ and $0 \le u\le t$; ${\mathcal {G}}$ is the augmented sigma algebra generated by $\bigcup _{t\ge 0}{\mathcal {G}}_t$. Likewise, for any $1\le i\le n$, ${\mathcal {F}}^i_t$ is the augmented natural filtration of $(D_u,P_u,\xi ^i_u)_{0\le u \le t}$, which represents the information of the *i*-th agent at time $t\ge 0$. (All augmentations are henceforth performed with the null sets of the sigma algebra ${\mathcal {G}}$.) Thus, for any $t\ge 0$ the objective of the *i*-th agent is

1.5 $$\begin{aligned} \max _{(c,\theta )\in {\mathcal {U}}^i}E\left[ \int _t^{\infty }e^{-\beta _i (u-t)}U^i(c_u)du\Big |{\mathcal {F}}^i_t\right] , \end{aligned}$$

where for any $1\le i\le n$, $U^i(c):=-\frac{e^{-\alpha _i c}}{\alpha _i}$, while the class of admissible consumption-investment strategies ${\mathcal {U}}^i$ is described in Definition 1.3 below.

To study each agent’s consumption-investment problem, we specify the functional form of the price of the risky asset with parameters to be determined in equilibrium. If the state of the economy were known to all agents, in view of the properties of exponential utility, it would be natural to guess that prices are affine in the state variables, i.e.,

1.6 $$\begin{aligned} P_t=C+\varepsilon _D D_t+\varepsilon _\pi \pi _t . \end{aligned}$$

However, each agent has only incomplete information about the state of the economy, summarized by the signals $\xi ^i$, and resulting in the individual (market) estimates $({\hat{\pi }}^i_t)_{t\ge 0}=(E[\pi _t|{\mathcal {F}}^i_t])_{t\ge 0}$. As the equilibrium price aggregates all individual signals, we replace $\pi _t$ in (1.6) by an estimate $\pi ^w_t$ that combines such information.

#### Definition 1.1

(i) Let $w_i>0$ ($1\le i \le n$). The *consensus estimate* $\pi ^w=(\pi ^w_t)_{t\ge 0}$ of the state of the economy with weights $w=(w_i)_{1\le i\le n}$ is defined as
  1.7 $$\begin{aligned} \pi _t^w:=E\left[ \pi _t\Bigg | \left( D_u,\sum _{i=1}^n w_i \xi ^i_u\right) _{0\le u \le t}\right] . \end{aligned}$$
(ii) A *linear price* is a process
  1.8 $$\begin{aligned} P_t=C+\varepsilon _D D_t+\varepsilon _\pi \pi _t^w, \quad t\ge 0, \end{aligned}$$
  where $\pi _t^w$ is the consensus estimate with weights $w=(w_i)_{1\le i\le n}$ and $C, \varepsilon _D, \varepsilon _\pi \in {\mathbb {R}}$.
(iii) Define the constants
  1.9 $$\begin{aligned} \nu :=\sigma _D^{-2}+\frac{(\sum _{i=1}^n w_i)^2}{\sum _{i=1}^n w_i^2\sigma _i^2}, \quad o_M :=\frac{-a+\sqrt{a^2+\sigma _\pi ^2\nu }}{\nu } \end{aligned}$$
  as well as
  1.10 $$\begin{aligned} \sigma _P^2:=\varepsilon _D^2\sigma _D^2+2\varepsilon _D\varepsilon _\pi o_M+\varepsilon _\pi ^2o_M^2\nu . \end{aligned}$$

#### Remark 1.2

(i) Because $D_0$ is deterministic and the signals $\xi$ are initially zero, for any weights $(w_i)_{1\le i\le n}$, any consensus estimate is initially $\pi _0^w={\bar{\pi }}$. As a further consequence, for any $1\le i\le n$,
  $$\begin{aligned} {\hat{\pi }}_0^i={\mathbb {E}}[\pi _0\mid {\mathcal {F}}_0^i]={\mathbb {E}}[\pi _0\mid \sigma (D_0,P_0)]={\mathbb {E}}[\pi _0\mid \sigma (D_0,\pi _0^w)]={\mathbb {E}}[\pi _0]={\bar{\pi }}. \end{aligned}$$
(ii) Lemma A.4 below justifies the notation $\sigma _P$ for the constant in (1.10) by showing that $\sigma ^2_P$ is indeed the squared volatility of a linear price (1.8) for a stationary Kalman-Bucy filter of the state of the economy.

#### Definition 1.3

(Admissible strategies) The set ${\mathcal {U}}^i$ of admissible strategies for the *i*-th agent is the set of all consumption-investment strategies $(c_t,\theta _t)_{t \ge 0}$ that satisfy the conditions:

(i) $(c_t)_{t \ge 0}$ and $(\theta _t)_{t \ge 0}$ are $(\mathcal F^i_t)_{t\ge 0}$-adapted processes such that $\mathbb E\left[ \int _0^T\vert \theta _t\vert ^2dt\right] ,\mathbb E\left[ \int _0^T\vert c_t\vert dt\right] <\infty$ for all $T>0$.
(ii) The wealth $X_t$ is self-financing, i.e.,
  1.11 $$\begin{aligned} dX_t=-c_t dt+\theta _t D_t dt+r(X_t-\theta _t P_t)dt+\theta _t dP_t,\qquad X_0=x^i_0, \end{aligned}$$
  and satisfies the transversality condition
  1.12 $$\begin{aligned} \limsup _{T\rightarrow \infty }\frac{\log {\mathbb {E}}[\vert X_T\vert ^2\mid {\mathcal {F}}_t^i]}{2T}< r-\frac{1}{2}r^2\vert {\bar{\alpha }}\vert ^2 \sigma _P^2, \end{aligned}$$
  where $${\bar{\alpha }}:=(\sum _{j=1}^n\frac{1}{\alpha _j})^{-1}$$.

#### Definition 1.4

A linear equilibrium in the economy is comprised of:

(i) A linear price as in (1.8), for some *C*, $\varepsilon _D$, $\varepsilon _\pi$, and $w=(w_i)_{i=1}^n$.
(ii) Optimal consumption-investment strategies $(c_t^i,\theta _t^i)_{t \ge 0}^{1\le i\le n}$ that clear the market, i.e.,
  1.13 $$\begin{aligned} \sum _{i=1}^n\theta ^{i}_t&=1 \qquad \text {a.s. for all }t\ge 0 . \end{aligned}$$

With the above definitions, it is now possible to state the main result of the paper, which characterizes the linear equilibrium in the model with heterogeneous information. The main result holds under two conditions: the first condition (1.14) ensures that the equilibrium is stationary, and is essentially equivalent to assuming that the market has been in existence for some time. The second condition (1.15) guarantees that the optimal trading strategy is admissible, as for the latter the limit on the left side of (1.12) is zero, while the right-hand side is strictly positive by assumption. The latter condition is satisfied, for instance, when the aggregate risk-aversion level ${\bar{\alpha }}$ is sufficiently small.

#### Theorem 1.5

Assume that

1.14 $$\begin{aligned} {\overline{\sigma }}_\pi ^2=o_M^\star =\frac{-a+\sqrt{a^2+\sigma _\pi ^2\nu ^\star }}{\nu ^\star }, \qquad \text {where}\qquad \nu ^\star =\sigma _D^{-2}+\sum _{j=1}^n \sigma _j^{-2}, \end{aligned}$$

and

1.15 $$\begin{aligned} \frac{r\vert {\bar{\alpha }}\vert ^2}{2}\left[ \left( \frac{\sigma _D}{k+r}+\frac{o_M^\star }{(a+r)(k+r)\sigma _D}\right) ^2+\frac{(o_M^\star )^2 (\nu ^\star -\sigma _D^{-2})}{(a+r)^2(k+r)^2}\right] <1 . \end{aligned}$$

Then, defining the constants:

1.16 $$\begin{aligned} w_i^\star =\sigma _i^{-2}, \quad \varepsilon _D^\star =\frac{1}{k+r}, \quad \varepsilon _\pi ^\star =\frac{1}{(k+r)(a+r)}, \quad C^\star =\frac{a{\bar{\pi }}}{r (k+r)(a+r)}-{\bar{\alpha }} \sigma _P^2,\nonumber \\ \end{aligned}$$

(i) The price of the risky asset, $P=(P_t)_{t\ge 0}$ of the form (1.8), where the market consensus (i) has weights $w_i=w_i^\star$, and $\varepsilon _D=\varepsilon _D^\star$, $\varepsilon _\pi =\varepsilon _\pi ^\star$, $C=C^\star$ (defined in (1.16)), is a linear equilibrium. The squared volatility (1.10) of *P* equals
  1.17 $$\begin{aligned} (\sigma _P^\star )^2=\frac{ \sigma _D^2}{(k+r)^2}+\frac{\sigma _\pi ^2}{(a+r)^2(k+r)^2}\left( 1+\frac{2 r}{a+\sqrt{a^2 +\sigma _\pi ^2(\frac{1}{\sigma _D^{2}}+\sum _{i=1}^n \frac{1}{\sigma _i^{2}})}}\right) .\nonumber \\ \end{aligned}$$
(ii) Under such equilibrium, the optimal strategy of the *i*-th agent ($1\le i\le n$) is
  1.18 $$\begin{aligned} c^{i}_t=\frac{\beta _i-r}{r\alpha _i}+r\left( X_t^i+\frac{{\bar{\alpha }}^2}{2\alpha _i}(\sigma _P^\star )^2\right) , \qquad \theta ^{i}_t=\frac{{\bar{\alpha }}}{\alpha _i}, \end{aligned}$$
  where $X_t^i$ denotes the wealth of the *i*-th agent.
(iii) The value function of the *i*-th agent ($1\le i\le n$) is
  1.19 $$\begin{aligned} {\mathbb {E}}\left[ \int _t^\infty e^{-\beta _i u} U^i(c^i_u)du\left| \right. {\mathcal {F}}_t^i\right] =\frac{-e^{-r\alpha _i X_t^i+\delta _0^i}}{r\alpha _i}, \quad \delta _0^i := -\frac{\beta _i-r}{r}-\frac{r{\bar{\alpha }}^2}{2}(\sigma _P^\star )^2.\nonumber \\ \end{aligned}$$
(iv) The market consensus is a positively recurrent process with dynamics
  $$\begin{aligned} d\pi _t^w=a({\overline{\pi }}-\pi _t^w)dt+{\hat{\sigma }}_\pi dZ_t,\quad \pi _0^w={\overline{\pi }}, \end{aligned}$$
  where $Z=(Z_t)_{t\ge 0}$ is a $({\mathcal {F}}^i_t)_{t\ge 0}$- standard Brownian motion for any $1\le i \le n$, and the volatility of the consensus estimate is^4^
  1.20 $$\begin{aligned} {\hat{\sigma }}_\pi =o_M^\star \sqrt{\nu ^\star } = \frac{-a+\sqrt{a^2+\sigma _\pi ^2\nu ^\star }}{\sqrt{\nu ^\star }} . \end{aligned}$$

#### Remark 1.6

The equilibrium in Theorem 1.5 is in fact unique up to scaling among linear equilibria, i.e., those of the form (1.8). Thus, uniqueness holds under a normalization condition such as $\sum _{i=1}^n w_i=1$ or $\sum _{i=1}^n w_i^2\sigma _i^2=\sum _{i=1}^n w_i$ (cf. Assumption A.5 below).

The proof of such uniqueness relies on lengthy but standard stochastic control arguments, whereby the value function of any linear equilibrium is found to be of exponential-quadratic form, hence the optimal number of shares is linear in the state variables. Aggregating asset demand, it follows that the only parameters that are compatible with the market clearing condition are those in Theorem 1.5. These details are not reported here for brevity, but are found in [5].

### Three equilibria

To understand the partial-information equilibrium quantities identified in this theorem, it is useful to compare them with two limit cases: (i) full-information and (ii) dividend-only.

Full-information equilibrium occurs when at least one agent’s signal becomes infinitely precise ($\sigma _i\downarrow 0$ for some *i*), and the knowledge of $\pi _t$ thus propagates to other agents through market prices. Indeed, as any $\sigma _i$ vanishes, (1.14) implies that $\nu ^\star$ diverges, hence ${\hat{\sigma }}_\pi$ in (1.20) converges to $\sigma _\pi$, and ${\overline{\sigma }}_\pi ^2$, the variance of the initial market state, converges to zero. The SDE for the market consensus (Theorem 1.5 (iv)) shows that, as $\sigma _i\downarrow 0$, the consensus estimate $\pi ^w$ converges in distribution to the true value of the state $\pi$. Likewise, price volatility in (1.17) simplifies to

1.21 $$\begin{aligned} (\sigma _P^\star )^2=\frac{ \sigma _D^2}{(k+r)^2}+\frac{\sigma _\pi ^2}{(a+r)^2(k+r)^2} . \end{aligned}$$

Vice versa, if all agents’ signals become infinitely imprecise ($\sigma _i\uparrow \infty$ for all *i*), the consensus estimate collapses to the estimate obtained by the dividend flow alone, because $\nu ^\star$ in (1.14) reduces to $\sigma _D^{-2}$. Thus the consensus estimate $\pi _t^w$ coincides with the conditional expectation (i.e., linear filter) of $\pi _t$ given $(D_s)_{s\le t}$, with volatility ${\hat{\sigma }}_\pi = \frac{-a+\sqrt{a^2+\sigma _\pi ^2/\sigma _D^2}}{\sigma _D^{-1}}$. Accordingly, price volatility in (1.17) becomes

1.22 $$\begin{aligned} (\sigma _P^\star )^2=\frac{ \sigma _D^2}{(k+r)^2}+\frac{\sigma _\pi ^2}{(a+r)^2(k+r)^2}\left( 1+\frac{2 r}{a+\sqrt{a^2 + \frac{\sigma _\pi ^2}{\sigma _D^{2}}}}\right) , \end{aligned}$$

reflecting the increased uncertainty on fundamentals. Thus, the full-information and dividend-only equilibria are the two extremes between which the partial-information equilibrium considered in this paper lays.

#### Remark 1.7

A cumbersome but straightforward differentiation shows that left-hand side of the parametric restriction in (1.15) is increasing in each of the $\sigma _i$, which means that, if such parametric restriction holds in the dividend-only equilibrium (i.e., setting $\nu ^\star =\sigma _D^{-2}$), then it also holds in any partial-information equilibrium (that is, for any choice of $(\sigma _i)_{i=1}^n$, holding other parameters fixed). In other words, the dividend-only setting is essentially the worst-case for the transversality condition: the validity of (1.15) for dividends-only guarantees its validity also with partial and full information.

The comparison between the partial-information equilibrium and the dividend-only or full-information equilibria helps to evaluate the potential impact of regulations such as the “disclose or abstain from trading” principle embedded in SEC Rule 10b-5 in US securities law, whereby it is unlawful for individuals with a fiduciary duty to shareholders to trade on material nonpublic information.

In the context of the present model, the main result implies that disclosing information is essentially equivalent to allowing trading, as information propagates instantaneously through prices to all market participants. Such release of information has in turn the ostensibly desirable effect of minimizing volatility, as the magnitude of shocks is reduced to reflect only the news that is unexpected to all participants.

Yet, the unintended consequence of reducing volatility is to deplete its aggregate risk premium, which leads to a decrease in welfare. Consequently, in this model agents would be best served by a “*do not* disclose *and* abstain from trading” policy, which would lead to higher volatility but also higher expected returns and welfare. As the next section shows, this effect is most pronounced for less risk-averse agents, who hold most of the asset (and earn its returns) in equilibrium.

## Implications

### Information in equilibrium

In the present model, the equilibrium endogenously identifies the consensus estimate $\pi ^{w}$ revealed by the market price $P_t$ as $\pi ^{w}_t = (P_t-C-\varepsilon _D D_t)/\varepsilon _\pi$. In particular, it is possible to understand the extent to which market prices aggregate and reveal the information of individual agents, by comparing the consensus estimate $\pi ^{w}$ to the estimate of a hypothetical omniscient agent who could observe all the signals $(\xi ^i)_{1\le i \le n}$ in addition to the dividend, i.e.,

$$\begin{aligned} \pi ^O_t = E\left[ \pi _t \left| \sigma \left( (D_u)_{0\le u\le t}, (\xi ^i_u)^{1\le i\le n}_{0\le u\le t}\right) \right. \right] . \end{aligned}$$

The calculation of $\pi ^O_t$ follows from the filtering results of Liptser and Shiryaev [19, Theorem 10.3] and coincides with $\pi ^{ w}$. Thus, the dynamic equilibrium price reveals not all information available to agents, as the individual signal $\xi ^i_t$ remains visible only to the *i*-th agent, but all the information that is necessary to obtain the same estimate of the state of the economy that a hypothetical omniscient agent would be able to achieve. In this sense, the market equilibrium provides an efficient mechanism for information aggregation, in that the information revealed by prices optimally aggregates individual signals, with no need for agents to disclose their private information.

In the resulting equilibrium, public information from prices and dividends alone already incorporates the contributions of all private signals, which are not used directly by the agents. Yet, each of the private signals is critical to price formation, as it enters the public signal with a positive weight. Put differently, if the *i*-th agent decided not to observe the private signal, the equilibrium price would be the one where only the others are present (which is equivalent to assuming that $\sigma _i$ is infinite). Then, if the same agent decided to observe the signal, trading the risky asset would become optimal until its price had reached the equilibrium that reflects such signal. In general, equilibrium weights for signals are inversely proportional to the signals’ respective variances, i.e., directly proportional to the signals’ precisions.

Note that this model only includes rational agents and a fixed asset supply, and leads to a fully revealing equilibrium. By contrast, partial-revelation equilibria rely crucially on the presence of noise traders, either explicitly, as in the asymmetric-information model of Kyle [18] and its numerous extensions, or implicitly through a stochastic asset supply (for which noise traders are responsible), as in the model of Hellwig [16] and its derivatives.^5^

The present model deliberately excludes noise trading in order to avoid ambiguity on the attribution of the welfare loss identified in the paper: if noise were present, one could plausibly ascribe agents’ suffering to the information degradation due to noise rather than to the information enhancement due to their signal. In the absence of noise, such ambiguity disappears, and we can firmly establish the role of increased information in reducing welfare.

### Volatility

Theorem 1.5 identifies squared volatility – the rate of change in the quadratic variation of the price – as (1.17). To understand this expression, it is useful to consider separately its different contributions. The first term ${ \sigma _D^2}/{(k+r)^2}$ reflects the variability of the discounted dividend stream, and is present even with a constant state of the economy ($\sigma _\pi = 0$).

The second term ${\sigma _\pi ^2}/{(a+r)^2(k+r)^2}$ is due to the variability of the state of economy, and has a different discount rate because the state of the economy affects the growth rate of dividends rather than their levels. Note that this second term is present even when the state is observable ($\sigma _i \downarrow 0$ for some *i*), in which case the third term vanishes. The third term is the only one that is affected by the quality of information on the state of the economy. In particular, volatility increases as quality decreases ($\sigma _i$ increases) and as the state becomes more persistent (*a* decreases).

Interestingly, a low interest rate *r* is associated with a smaller impact of information quality. Upon reflection, this observation is consistent with the stationarity of the state of the economy: at long horizons, the state reverts to its long term mean, and information about the current state is less relevant. It is precisely when interest rates are low that prices reflect the risk-adjusted value of dividends at longer maturities, for which information on the current state of the economy has a lower impact.

Note also that the above formula implies that volatility is independent of agents’ preferences, which means that in this model an increase in risk aversion has no effect on volatility. As discussed next, the effect of risk aversion is on prices. Finally, the number of agents affects volatility only through the total signal precision $\sum _{i=1}^n \sigma _i^{-2}$. Put differently, a market with a single agent having a private signal with noise $\sigma _1$ is equivalent to a market with *n* agents having independent private signals with noise $\sigma _1 \sqrt{n}$.

### Prices

The above considerations on volatility are key to understand the dependence of prices on the model’s parameters. The price of the risky asset is

2.1 $$\begin{aligned} P_t =&\frac{a{\bar{\pi }}}{r(a+r)(k+r)}+\frac{1}{k+r} D_t+\frac{1}{(a+r)(k+r)}\pi ^{ w}_t -{\bar{\alpha }} \sigma _P^2 \nonumber \\ =&E\left[ \int _t^{\infty }e^{-r(u-t)}D_u du \left| \sigma \left( \left( D_u,\sum _{i=1}^n w_i \xi ^i_u\right) _{u \le t}\right) \right. \right] -{\bar{\alpha }} \sigma _P^2. \end{aligned}$$

Put differently, the first three terms (2.1) reflect the expected present value of future dividends using equilibrium information. By contrast, the last term $-{\bar{\alpha }} \sigma _P^2$ represents the price discount that arises in equilibrium from the aggregate risk aversion ${\bar{\alpha }}$ of the agents. Thus, while an increase in risk aversion does not affect volatility, it does reduce prices by increasing the discount below their expected present value.

A further message of the above equation is that information affects price levels only through (i) the present value of future dividends, and (ii) volatility. In particular, the estimate of the state of the economy $\pi ^{w}$ affects prices only through the present value of dividends, and does not affect the discount for risk, which remains constant over time. It also does not affect the sensitivity of prices to the current dividend $D_t$ and the state of the economy $\pi ^{ w}$.

### Welfare

The optimal consumption rate of the *i*-th agent is

2.2 $$\begin{aligned} c^{i\star }_t=\frac{\beta _i-r}{r\alpha _i}+r\left( X_t^{i\star }+\frac{ {\bar{\alpha }}^2}{2\alpha _i}\sigma _P^2\right) . \end{aligned}$$

The first term in this formula reflects the agent’s time preference and intertemporal substitution, and is constant. More impatient (higher $\beta _i$) and intertemporally inelastic (lower $\alpha _i$) agents consume more, regardless of the dynamics of dividends. (Note that, as the model assumes an exogenous interest rate *r*, aggregate consumption $\sum _{i=1}^n c^i_t$ does not necessarily match dividends $D_t$ because a safe asset in unlimited supply makes aggregate accumulation and depletion possible.)

An implication of (2.2) is that consumption depends on dividends and the state of the economy only through wealth: given the equilibrium price, the agent consumes as if the state of the economy were constant. Hence, agents do not have to use complicated consumption-investment policies to achieve a rational expectations equilibrium: even if they were able to only optimize among constant investment strategies and among consumption policies that are affine in wealth, they would still reach the same equilibrium.

To understand the second and third terms in (2.2), it is useful to observe that, from the formula for the value function (1.19), the *i*-th agent is indifferent between (i) starting with $X_t^{i\star }$ in cash and then live in the market where the risky asset is available, and (ii) living in a simpler market, where only the safe asset exists, but starting with the higher cash amount

2.3 $$\begin{aligned} {\bar{X}}^i_t = X_t^{i\star }+\frac{ {\bar{\alpha }}^2}{2\alpha _i} \sigma _P^2 , \end{aligned}$$

which thus represents the certainty equivalent of the *i*-th agent.

Hence, the second and third terms in (2.2) are interpreted as the rent that the agent collects from the certainty equivalent $X_t^{i\star }+\frac{ {\bar{\alpha }}^2}{2\alpha _i}\sigma _P^2$: it represents the minimum amount of money that at time *t* the agent would accept to give up the opportunity to invest in the risky asset. As it is natural, such certainty equivalent is proportional to the total discount ${\bar{\alpha }} \sigma _P^2$ of the risky asset below its risk neutral value, and to the number of shares $\theta ^i = {\bar{\alpha }}/\alpha _i$ in the agent’s portfolio.

In relation to information, the consumption formula (2.2) has a central message: as more information enters the market, in equilibrium all agents become worse off because their consumption declines. Although this result is superficially surprising, it is in fact consistent with the previous observations that information reduces volatility, which in turn reduces the price discount. Because the certainty equivalent is additive in the price discount, it follows that consumption has to decrease as volatility decreases.

This observation highlights a potential tension between informational and economic efficiency. In the present model, self-interested rational agents achieve anonymously the same informational efficiency as a hypothetical omniscient central planner. Yet, while each of them attempts to use private information to gain an edge on the others, the overall result is that all of them are worse off as a result, mimicking qualitatively the classical prisoner’s dilemma paradox. Note also that each of them could make everyone (including oneself) better off by foregoing one’s private signal in the decision process, thereby switching to an equilibrium with higher volatility, price discount, and hence consumption. But such a decision would require perfect commitment: otherwise, in the new equilibrium, the agent would be tempted to peek at the private signal to gain a temporary advantage over the others, eventually bringing prices back to the original equilibrium.

### New vs. old investors

The certainty equivalent formula (2.3) also helps to understand in which sense an increase in information benefits existing assets’ owners rather than investors, as mentioned in the introduction.^6^ Imagine that the *i*-th agent starts with an endowment of $c^i$ in cash and $\theta ^i_{0^-}$ shares. Thus, the certainty equivalent is

2.4 $$\begin{aligned} c^i + \theta ^i_{0^-} P_0 + \frac{ {\bar{\alpha }}^2}{2\alpha _i} \sigma _P^2 , \end{aligned}$$

where the additional middle term reflects the value of the shares in the endowment.

The agent’s intention is to immediately trade at time 0, as to hold the optimal number of shares ${\bar{\alpha }}/\alpha _i$. Now, suppose that the quality of information on the asset changes before the agent can trade (i.e., between $0-$ and 0), so that the variance $\sigma _P^2$ increases by $\Delta$. In view of (1.16), the agent’s welfare increases by

2.5 $$\begin{aligned} - \theta ^i_{0^-} {\bar{\alpha }}\Delta + \frac{ {\bar{\alpha }}^2}{2\alpha _i} \Delta = \left( \frac{{\bar{\alpha }}}{2\alpha _i} - \theta ^i_{0^-} \right) {\bar{\alpha }}\Delta . \end{aligned}$$

Thus, an improvement in information quality ($\Delta$ negative) results in a welfare decrease if the endowment is less than half of the optimal asset position ($\theta ^i_{0^-} < \frac{{\bar{\alpha }}}{2\alpha _i}$), otherwise in an increase.

The intuition is straightforward: if the endowment is only in cash, welfare increases with volatility through the earned risk premium, as observed above. However, if the endowment also includes shares, then the agent may also gain or lose from the effect of volatility on the asset price. Because the price increases as volatility decreases, if the agent’s position is sufficiently high, then the gain in price overrides the welfare loss from the lower risk premium earned in the future.

These remarks offer a clear rationale for company founders and early investors to improve information quality before taking a company public: regardless of regulatory requirements, it is in their personal interest to improve investors’ knowledge of their company’s fundamentals, as to increase its stock price. (While in the present model, which has a single risky asset, this effect is tantamount to a reduction in the variance of the company’s stock price, in general it would entail minimizing *systematic* risk, because idiosyncratic risk does not affect stock prices.)

Finally, note that a lower variance actually *increases* the welfare of the representative agent, who has risk aversion ${\bar{\alpha }} = 1/\sum _{i=1}^n \frac{1}{\alpha _i}$ and holds the whole asset (i.e., one share) both before and after any change in information quality. Indeed, (2.4) implies that a variation $\Delta$ in price variance leads to a change in the certainty equivalent equal to $(1/2 - 1){\bar{\alpha }}\Delta$. That is, welfare increases as variance decreases, but such an increase is entirely ascribed to previous owners, whose gains more than offset the losses of all new entrants. In this sense, the representative agent is only representative of the old, not the new, investors.

## Heuristics

In equilibrium, all agents must agree on the same price (otherwise some would want to trade). As prices should depend on dividends – which are public – and on an estimate of the state of the economy, it follows that agents should agree in equilibrium on a common estimate, i.e., the consensus. As exponential utility leads to affine demand functions in the states variables $D_t$ and $\pi ^w$, and the asset supply is fixed, it is also natural to guess that prices are affine in states, i.e., $P_t=C+\varepsilon _D D_t+\varepsilon _\pi \pi _t^w$. Denoting by $X_t^i$ the *i*-th agent’s wealth process, the self-financing condition implies

$$\begin{aligned} dX_t^i&= -c_t dt+\theta ^i_t D_t dt+r(X^i_t-\theta ^i_t P_t)dt+\theta ^i_t dP_t,\\&=- (c_t^i-rX_t) dt+\theta ^i_t D_t dt-r\theta _t^i P_t dt+\theta ^i_t (\varepsilon _D dD_t+\varepsilon _\pi d\pi ^w_t). \end{aligned}$$

Note that ${\hat{\pi }}^i$ and $\pi ^w$ are indistinguishable for $1\le i\le n$, therefore $w_i=w_i^\star =\sigma _i^{-2}$ for $1\le i\le n$ (Lemma A.8), therefore Lemma (iii) implies that

$$\begin{aligned} d\pi ^w_t&=a({\bar{\pi }} -\pi ^w_t)dt+o_M\left( \sigma _D^{-1} dB^{iD}_t+\sum _{j=1}^n\sigma _j^{-1} dB^{j}_t\right) ,\\ dD_t&=(\pi ^w_t-kD_t)dt+\sigma _D dB^{iD}_t. \end{aligned}$$

Denoting by $\nu ^\star$ and $o_M^\star$ the values of $\nu$ and $o_M$ obtained from $w_i^\star$, it follows that

$$\begin{aligned} dX_t^i= \mu _t^i dt+\theta _t^i\left( (\varepsilon _D\sigma _D+\varepsilon _\pi o_M^\star \sigma _D^{-1}) dB_t^{iD}+\varepsilon _\pi o_M^\star \sum _{i=1}^n \sigma _i^{-1}dB_t^{i}\right) , \end{aligned}$$

where

3.1 $$\begin{aligned} \mu _t^i=rX_t^i-c_t^i+\theta ^i_t D_t-r\theta _t^i (C+\varepsilon _D D_t+\varepsilon _\pi \pi ^w_t)+\theta _t^i(\varepsilon _D(\pi ^w_t-kD_t)+\varepsilon _\pi a({\bar{\pi }} -\pi ^w_t)).\nonumber \\ \end{aligned}$$

Note that the instantaneous quadratic variation is

3.2 $$\begin{aligned} \frac{d\langle X^i\rangle _t}{dt}=(\theta _t^i)^2\left( (\varepsilon _D\sigma _D+\varepsilon _\pi o_M^\star \sigma _D^{-1}) ^2+(\varepsilon _\pi o_M^\star )^2\sum _{i=1}^n \sigma _i^{-2}\right) =(\theta _t^i)^2\sigma _P^2. \end{aligned}$$

Next, consider the value function of the *i*-th agent. A priori, it may depend on the agent’s wealth, the dividend $D_t$, and the consensus estimate $\pi ^w_t$. Individual wealth is essential, but the question is whether the dependence on the other two variables is *separate* from wealth or only *through* wealth. It is natural to attempt the latter approach, as it implies a value function of the type $V^i=V^i(X^i)$ rather than a function of three states. The corresponding Hamilton-Jacobi-Bellman equation is

3.3 $$\begin{aligned} \sup _{c,\theta \in {\mathcal {U}}^i}\left( \frac{e^{-\alpha _i c^i_t}}{-\alpha _i}-\beta _i V^i+ \mu _t^i V_x^i +\frac{V^i_{xx}}{2}\frac{d\langle X^i\rangle _t}{dt} \right) =0. \end{aligned}$$

Consistently with the exponential utility, guessing $V^i(X^i)=\frac{e^{-r\alpha _i X^i+\delta _0^i}}{-r\alpha _i}$ and using equations (3.1)-(3.2), the first order conditions are

3.4 $$\begin{aligned} c_t^i=\frac{\log (V_x^i)}{-\alpha _i}=rX_t^i-\frac{\delta _0^i}{\alpha _i} \end{aligned}$$

for consumption and

3.5 $$\begin{aligned} \theta _t^i&=-\frac{V_x^i}{V_{xx}^i}\left( \frac{D_t(1-(k+r)\varepsilon _D) +\pi _t^w(\varepsilon _D-(a+r)\varepsilon _\pi )-rC+a{\bar{\pi }}\varepsilon _\pi }{\varepsilon _D^2\sigma _D^2+(o_M^\star )^2\varepsilon _\pi ^2 \nu ^\star +2\varepsilon _D\varepsilon _\pi o_M^\star }\right) \nonumber \\&=\frac{1}{r\alpha _i}\left( \frac{D_t(1-(k+r)\varepsilon _D)+ \pi _t^w(\varepsilon _D-(a+r)\varepsilon _\pi )-rC+a{\bar{\pi }} \varepsilon _\pi }{\varepsilon _D^2\sigma _D^2+(o_M^\star )^2\varepsilon _\pi ^2 \nu ^\star +2\varepsilon _D\varepsilon _\pi o_M^\star }\right) \end{aligned}$$

for investment. The market clearing condition (1.13) dictates that the coefficients of $D_t$, $\pi _t^w$ vanish, which imply the constants $\varepsilon _D=\varepsilon _D^\star$ and $\varepsilon _\pi =\varepsilon _\pi ^\star$ as stated in (1.16), and that

$$\begin{aligned} {\bar{\alpha }}^{-1}\frac{-C+\frac{a{\bar{\pi }}}{r}\varepsilon _\pi ^\star }{(\varepsilon _D^\star )^2\sigma _D^2+(o_M^\star \varepsilon _\pi ^\star )^2 \nu ^\star +2\varepsilon _D^\star \varepsilon _\pi ^\star o_M^\star }=1. \end{aligned}$$

Elementary algebraic manipulations yield (1.17) for the squared volatility $(\sigma _P^*)^2$ of the equilibrium price *P*. Thus, the market clearing condition implies that

$$\begin{aligned} {\bar{\alpha }}^{-1}\frac{-C+\frac{a{\bar{\pi }}}{r}\varepsilon _\pi ^\star }{(\sigma _P^\star )^2}=1, \end{aligned}$$

whence the constant $C=C^\star$ as in (1.16). Then, equation (3.5) yields the candidate optimal trading policy for the *i*-th agent,

$$\begin{aligned} \theta ^i_t=\frac{{\bar{\alpha }}}{\alpha _i}, \end{aligned}$$

which is the second formula in (1.18). It remains to compute the constant $\delta _0^i$, which in turn identifies both the value function and the optimal consumption. Inserting $\theta ^i$ and $c_t^i$ from (3.4) into the HJB equation (3.3) yields (1.19) which, again after some algebraic manipulations, obtains the consumption formula in (1.18).

## Conclusion

This paper investigates the aggregation of disperse information in a financial market with rational agents who maximize lifetime utility from consumption, learning from both public prices and private signals. The market aggregates information optimally because the resulting consensus estimate of the state of the economy is the same as the one that a hypothetical agent with access to all private information could obtain.

In equilibrium, more information reduces price volatility, and in particular the component that stems from uncertainty on the state of the economy in the near future. Such an effect is more pronounced when interest rates are higher, and therefore the relative weight of near-term dividends is higher. However, volatility mitigation does not translate into higher utility for market participants because its primary effect is to more closely align asset prices with the present value of their dividends, thereby reducing the risk premium.

Thus, market participants find themselves in a predicament, whereby each of them uses private information to make optimal investment and consumption decisions, but the net effect is that the useful component of such information is revealed to other participants through prices, and everyone earns a lower risk premium in the future as a result of informational efficiency.

## Data Availability

Data sharing not applicable to this article as no datasets were generated or analysed during the current study.

## A. Proofs

### A.1 Filtering results

Applying [19, Theorem 10.3] to the setup of this paper, the Kalman-Bucy filter can be stated as:

#### Theorem A.1

Let $(W^\pi ,W)$ be a $k+1$-dimensional ($k\ge 1$) standard Brownian motion (with $W^\pi$ being one-dimensional) and $a_0\in {\mathbb {R}}$, $A_1 \in {\mathbb {R}}^k$ and $A_2,B$ be $k\times k$ real-valued matrices. Suppose $a_1<0$ and $b\in {\mathbb {R}}\setminus \{ 0\}$. Consider the one-dimensional process $(\Pi _t)_{t\ge 0}$ and the three-dimensional process $(\Psi _t)_{t\ge 0}$ with dynamics

$$\begin{aligned} d\Pi _t&=(a_0+a_1 \Pi _t)dt+b dW^\pi _t,\\ d\Psi _t&=(A_1\Pi _t+A_2 \Psi _t)dt+B dW_t, \end{aligned}$$

such that $\Psi _0\in {\mathbb {R}}^k$, and $\Pi _0\sim {\mathcal {N}}({\bar{\Pi }},\kappa _0)$ is normally distributed, independent of $(W^\pi , W)$, where $\kappa _0>0$. Denote by $({\mathcal {G}}_t)_{t\ge 0}$ the filtration generated by $(\Psi _t)_{t\ge 0}$, and let $\kappa (t)$ be the unique non-negative solution of the Riccati differential equation

A.1 $$\begin{aligned} {\dot{\kappa }}=2a_1\kappa +b^2-\kappa ^2 (A_1^\top (B B^\top )^{-1} A_1)=0,\quad \kappa (0)=\kappa _0. \end{aligned}$$

Then the Kalman-Bucy filter $({\hat{\Pi }}_t)_{t\ge 0}$ (where ${\hat{\Pi }}_t:={\mathbb {E}}[\Pi _t\mid {\mathcal {G}}_t]$) of the process $(\Pi _t)_{t\ge 0}$ with signal $(\Psi _t)_{t\ge 0}$ is the unique solution of the stochastic differential equation

$$\begin{aligned} d{\hat{\Pi }}_t=(a_0+a_1 {\hat{\Pi }}_t)dt+\kappa (t) A_1^\top (BB^\top )^{-1}[d\Psi _t-(A_1{\hat{\Pi }}_t+A_2 \Psi _t)dt],\quad {\hat{\Pi }}_0={\bar{\Pi }}, \end{aligned}$$

and $\int _0^\cdot (BB^\top )^{-1/2}[d\Psi _t-(A_1{\hat{\Pi }}_t+A_2 \Psi _t)dt]$ is a $({\mathcal {G}}_t)_{t\ge 0}$- Brownian motion. Furthermore, $\kappa (t)$ is the mean-square error^7^ of the prediction, that is,

$$\begin{aligned} \kappa (t)={\mathbb {E}}[({\hat{\Pi }}_t-\Pi _t)^2]. \end{aligned}$$

In particular, if $\kappa _0$ is the unique solution of the algebraic Riccati equation

A.2 $$\begin{aligned} 2a_1\kappa _0 +b^2-\kappa _0^2 (A_1^\top (B B^\top )^{-1} A_1)=0, \end{aligned}$$

then the Kalman-Bucy filter is stationary in the sense that $\kappa (t)\equiv \kappa _0$ for $t\ge 0$.

#### Remark A.2

Note that the initial condition of (A.1) is $\text {Var}(\Pi _0-{\mathbb {E}}[\Pi _0\mid \Psi _0])=\text {Var}(\Pi _0)=\kappa _0$ because $\Psi _0$ is deterministic.

#### Proof of Theorem A.1

To apply [19, Theorem 10.3], set therein $\theta =\Pi$, $\xi =\Psi$, and furthermore $W_1=W^\pi$ and $W_2=W$, leading to the values

(i) (drift coefficients) $a_2=0$, $A_0=0$, and $a_0, a_1$, as well as $A_1, A_2$ are constants,
(ii) (diffusion coefficients) $b_2\equiv 0$, and $B_1\equiv 0$, while $b_1(t)\equiv b$ and $B_2(t)\equiv B$ are constants.

Therefore, the definitions in [19, (10.8)] simplify to the following (constant) expressions

$$\begin{aligned} (b\circ b)(t)\equiv b_1 b_1^\top ,\quad (b\circ B)(t)\equiv 0\quad \text {and}\quad (B\circ B)(t)\equiv B_2 B_2^\top . \end{aligned}$$

Thus, by [19, Theorem 10.3], $m_t={\hat{\Pi }}_t$ satisfies

$$\begin{aligned} d{\hat{\Pi }}_t=(a_0+a_1 {\hat{\Pi }}_t)dt+\kappa (t) A_1^\top (BB^\top )^{-1}[d\Psi _t-(A_1{\hat{\Pi }}_t+A_2 \Psi _t)dt],\quad {\hat{\Pi }}_0={\mathbb {E}}[\Pi _0\mid {\mathcal {G}}_0], \end{aligned}$$

where the mean-square error of the prediction equals $\kappa (t)>0$, which is the unique non-negative solution of (A.1).

If, in addition, the variance of ${\hat{\Pi }}_0$ equals the unique positive root of (A.2), then clearly $\kappa (t)\equiv \kappa$, and the second claim follows. $\square$

#### Lemma A.3

The consensus estimate $(\pi ^w_t)_{t\ge 0}$ of the state of the economy has dynamics

A.3 $$\begin{aligned} d\pi ^w_t&= \left[ a({\bar{\pi }}-\pi ^w_t)+ o_M(t) k\sigma _D^{-2} D_t-o_M(t)\left( \sigma _D^{-2}+ \frac{(\sum _{i=1}^n w_i)^2}{\sum _{i=1}^n w_i^2 \sigma _i^2}\right) \pi ^w_t\right] dt\nonumber \\&+o_M(t)\left( \sigma _D^{-2} dD_t+\frac{\sum _{i=1}^n w_i}{\sum _{i=1}^n w_i^2 \sigma _i^2}\sum _{i=1}^n w_i d\xi ^i_t\right) , \end{aligned}$$

where $\pi ^w_0 = {\bar{\pi }}$ and $o_M(t)$ satisfies the Riccati differential equation

$$\begin{aligned} \dot{o}_M(t)=-2 a o_M(t)+\sigma _\pi ^2-\nu o_M(t)^2,\quad o_M(0)={\overline{\sigma }}_\pi ^2. \end{aligned}$$

In particular, if ${\overline{\sigma }}^2_\pi =o_M$, then $o_M(t)\equiv o_M={\overline{\sigma }}^2_\pi$, for all $t\ge 0$.

#### Proof

Apply Theorem A.1 with the processes $(D_t,\sum _{i=1}^n w_i \xi ^i_t)_{t\ge 0}$ as signals. $\square$

#### Lemma A.4

If ${\overline{\sigma }}_\pi ^2=o_M$, then the squared volatility of the linear price (1.8) is as in (1.10).

#### Proof

By Lemma (iii) A.7, ${\overline{\sigma }}_\pi ^2=o_M$ implies that $o_M(t)\equiv o_M$. From the system of SDEs A.8, it follows that

$$\begin{aligned} \sigma _p^2=\left( \varepsilon _D^2\sigma _D^2+\varepsilon _\pi ^2o_M^2 \sigma _D^{-2}+2\varepsilon _D\varepsilon _\pi o_M\right) +\varepsilon _\pi ^2 o_M^2\left( \frac{(\sum _{i=1}^n w_i)^2}{\sum _{i=1}^n w_i^2\sigma _i^2}\right) . \end{aligned}$$

which equals (1.10) by by (1.9). $\square$

#### Assumption A.5

For convenience of notation, in the remainder of this section assume

A.4 $$\begin{aligned} \sum _{i=1}^n w_i^2\sigma _i^2=\sum _{i=1}^n w_i. \end{aligned}$$

This assumption entails no loss of generality because $(\pi ^w_t)_{t\ge 0}$ is invariant with respect to a common scaling factor $\lambda >0$, i.e., $\pi ^{\lambda w}$ is indistinguishable from $\pi ^{w}$. To wit, for any vector of weights *w*, it suffices to normalize the weights to $\lambda w$, where $\lambda = \sum _{i=1}^n w_i/\sum _{i=1}^n w_i^2 \sigma _i^2$. Note that by (A.4), the parameter defined in (1.9) may be written in two equivalent forms,

$$\begin{aligned} \nu =\sigma _D^{-2}+\sum _{i=1} w_i=\sigma _D^{-2}+\sum _{i=1} w_i^2\sigma _i^2. \end{aligned}$$

Also, note that (A.3) simplifies now to

A.5 $$\begin{aligned} d\pi ^w_t&= \left[ a({\bar{\pi }}-\pi ^w_t)+ o_M(t) k\sigma _D^{-2} D_t-o_M(t)\left( \sigma _D^{-2}+\sum _{i=1}^n w_i\right) \pi ^w_t\right] dt\nonumber \\&\quad +o_M(t)\left( \sigma _D^{-2} dD_t+\sum _{i=1}^n w_i d\xi ^i_t\right) , \end{aligned}$$

The rest of this section relates the individual estimates of each agent $({\hat{\pi }}^i_t)_{t\ge 0}$ to the consensus estimate $(\pi ^w_t)_{t\ge 0}$. To this end, we introduce further constants

#### Definition A.6

Recalling $\nu$ and $o_M$ from Definition 1.1 (iii), define the constants

$$\begin{aligned}&w_{i \perp } :=\sqrt{\sum _{j\ne i} w_j^2 \sigma _j^2},&\sigma _{i\perp } :=\frac{\sqrt{\sum _{j\ne i} w_j^2 \sigma _j^2}}{\sum _{j\ne i} w_j},\\&\nu _i :=\sigma _D^{-2}+\sigma _i^{-2}+\sigma _{i\perp }^{-2},&o_i :=\frac{-a+\sqrt{a^2+\sigma _\pi ^2\nu _i}}{\nu _i}. \end{aligned}$$

We start by finding the agents’ views about the state of the economy.

#### Lemma A.7

(Filtering) Define

$$\begin{aligned} \xi _t^{i\perp }&:=\frac{1}{\sum _{j\ne i} w_j}\sum _{j\ne i} w_j \xi _t^j, \qquad {\hat{\pi }}^i_t:=E[\pi _t|{\mathcal {F}}^i_t], \end{aligned}$$

and the stochastic processes $B:=(B_t)_{t\ge 0}=(B^{iD}_t,B^{i}_t,B^{i\perp }_t)_{t\ge 0}$, where

A.6 $$\begin{aligned} B_t^{iD}&:=W_t^D+\int _0^t\frac{\pi _u-{\hat{\pi }}^i_u}{\sigma _D}du,\qquad B^i_t:=W_t^i+\int _0^t\frac{\pi _u-{\hat{\pi }}^i_u}{\sigma _i}du,\nonumber \\ B^{i\perp }_t&:=\frac{1}{w_{i\perp }}\sum _{j\ne i} w_j \sigma _j W_t^{j}+ \int _0^t\frac{\pi _u-{\hat{\pi }}^i_u}{\sigma _{i\perp }}du. \end{aligned}$$

The following hold:

(i) For every $$t\ge 0$$, $$ {\mathcal {F}}^i_t=\sigma \{D_u, \pi ^w_u, \xi ^i_u\}_{0 \le u \le t}=\sigma \left\{ D_u, \xi _u^{i\perp }, \xi ^i_u\right\} _{0 \le u \le t}$$.
(ii) The *i*-th agent’s (stationary) filter for the state of the economy is
  A.7 $$\begin{aligned} d{\hat{\pi }}^i_t=a({\bar{\pi }} -{\hat{\pi }}^i_t)dt+o_i(t)\left( \sigma _D^{-1}dB^{iD}_t+\sigma _i^{-1}dB^i_t+\sigma _{i\perp }^{-1}dB^{i\perp }_t\right) , \quad {\hat{\pi }}^i_0={\bar{\pi }}, \end{aligned}$$
  *B* is a standard $({\mathbb {P}},{\mathcal {F}}_t^i)_{t\ge 0})$-Brownian motion, and $o_i(t)$ satisfies the Riccati differential equation
  $$\begin{aligned} \dot{o}_i(t)=-2 a o_i(t)+\sigma _\pi ^2-\nu _i o_i(t)^2 ,\quad o_i(0)={\overline{\sigma }}_\pi ^2. \end{aligned}$$
  In particular, if ${\overline{\sigma }}^2_\pi =o_i$, then $o_i(t)\equiv o_i={\overline{\sigma }}^2_\pi$, for all $t\ge 0$.
(iii) For every $1\le i\le n$, the processes $(\pi ^w_t)_{t\ge 0}$ and $(D_t)_{t\ge 0}$ follow the dynamics
  A.8 $$\begin{aligned} d\pi ^w_t&= [a({\bar{\pi }} -\pi ^w_t)+o_M(t) \nu (\pi ^i_t-\pi ^w_t)]dt\nonumber \\&\quad +o_M(t)\left( \sigma _D^{-1}dB^{iD}_t+w_i\sigma _i dB^i_t+w_{i\perp } dB^{i\perp }_t\right) , \nonumber \\ dD_t&= ({\hat{\pi }}^i_t-kD_t)dt+\sigma _D dB^{iD}_t, \end{aligned}$$
  where $o_M(t)$ satisfies the Riccati differential equation
  $$\begin{aligned} \dot{o}_M(t)=-2 a o_M(t)+\sigma _\pi ^2-o_M(t)^2 (\sigma _D^{-2}+\sum _{i=1}^n w_i),\quad o_M(0)={\overline{\sigma }}_\pi ^2. \end{aligned}$$
  In particular, if ${\overline{\sigma }}^2_\pi =o_M$, then $o_M(t)\equiv o_M={\overline{\sigma }}^2_\pi$, for all $t\ge 0$.

#### Proof

Define ${\mathcal {H}}^i_t=\sigma \{D_u,\pi ^w_u, \xi ^i_u: 0 \le u \le t\}$ and ${\mathcal {L}}^i_t=\sigma \left\{ D_u, \sum _{j\ne i} w_j \xi ^j_u, \xi ^i_u: 0 \le u \le t\right\}$. Equation (1.8) implies $({\mathcal {F}}^i_t)_{t\ge 0}=({\mathcal {H}}^i_t)_{t\ge 0}$.

Proof of ${\mathcal {H}}^i_t \subseteq {\mathcal {L}}^i_t$: As *D* and $\xi ^i$ are ${\mathcal {L}}^i$-adapted, by (A.5), also $\pi ^w$ is adapted to the filtration generated by $D,\xi ^i$ and $\xi ^{i,\perp }$.

Proof of ${\mathcal {L}}^i_t \subseteq {\mathcal {H}}^i_t$: Dividing (A.5) by $o_M(t)$, and integrating by parts allows to write $\xi ^{i\perp }$ as a non-anticipating functional of the paths of $\xi ^{i}$, $\pi ^w$ and *D*. Hence $\xi ^{i\perp }$ is ${\mathcal {H}}^i$-adapted.

Applying Theorem A.1 with the process $(D_t,\xi ^i_t,\xi ^{i\perp }_t)_{t\ge 0}$ as signal, A.7 follows, while A.7 is a direct consequence of A.7, Lemma A.3 and the definition A.7 of the Brownian motion $(B^{iD}_t,B^{i}_t,B^{i\perp }_t)_{t\ge 0}$. $\square$

The market estimate of the state of the economy reveals a weighted average of the private information available. The (second part of the) following Lemma shows that each agent considers $(\pi ^w_t)_{t\ge 0}$ the best approximation for $(\pi _t)_{t\ge 0}$ if the weight of the private signal in the process $(\pi _t^w)_{t\ge 0}$ is the inverse of the square of the signal’s noise, i.e. $w_i=\sigma _i^{-2}$.

#### Lemma A.8

(Properties of filters)

(i) Let $\sigma _\pi >0$ and $1\le i\le n$. The following are equivalent:
  - The $({\mathcal {G}}_t)_{t\ge 0}$-measurable processes $(\pi _t^w)_{t\ge 0}$ and $({\hat{\pi }}^i_t)_{t\ge 0}$ are indistinguishable.
  - $w_i=\sigma _i^{-2}$, $\nu _i=\nu$ (or, equivalently, for all $t\ge 0$, $o_i(t)=o_M(t)$) and $\sigma _{i\perp }^{-1}=w_{i\perp }$.
(ii) If $w_i=\sigma _i^{-2}$ for all $1\le i\le n$, then ${\hat{\pi }}^i_t = \pi ^w_t$ a.s. for all $t\ge 0$ and $1\le i\le n$.

#### Proof

The statement A.8 follows from A.8. The characterization in A.8 holds in view of the explicit SDEs for the consensus estimate and the individual estimates, (A.8) and (A.7). $\square$

### Existence

This section shows the existence of a linear equilibrium, as stated in Theorem 1.5. First, note that the market clears for the stated strategies because, by the definition of ${\bar{\alpha }}$,

$$\begin{aligned} \sum _{i=1}^n \theta ^i_t={\bar{\alpha }}\sum _{i=1}^n\frac{1}{\alpha _i}=1 . \end{aligned}$$

Therefore, it remains to show that each agent acts optimally, given the stated price process. In accordance with the assumption of Theorem 1.5 we assume throughout this section that the constants $C=C^\star ,\varepsilon _D=\varepsilon _D^\star$ and $\varepsilon _\pi =\varepsilon _\pi ^\star$ as given by (1.16), that $w_i=w_i^\star =\sigma _i^{-2}$ for $1\le i\le n$, and that ${\overline{\sigma }}_\pi ^2=o_M^\star$. (The latter condition implies stationarity of the filter, see Lemma (iii).)

#### Definition A.9

A stochastic discount factor (SDF) for the *i*-th agent is a positive, continuous, $({\mathcal {F}}^i_t)_{t\ge 0}$-adapted process $({\mathcal {H}}^i_t)_{t\ge 0}$ such that ${\mathcal {H}}^i_0=1$, ${\mathbb {P}}$-almost surely, and for every $0\le s\le t$

A.9 $$\begin{aligned} {\mathcal {H}}^i_s e^{rs}=E[e^{rt}{\mathcal {H}}^i_t |{\mathcal {F}}^i_s], \end{aligned}$$

as well as

A.10 $$\begin{aligned} {\mathcal {H}}^i_s P_s+\int _0^s {\mathcal {H}}^i_u D_u du=E\left[ {\mathcal {H}}^i_t P_t+\int _0^t {\mathcal {H}}^i_u D_u du \Big |{\mathcal {F}}^i_s\right] . \end{aligned}$$

Consider now the stochastic discount factors, for $1\le i\le n$,

A.11 $$\begin{aligned} {\mathcal {N}}^i_t= & {} \exp \Big (-rt+\int _0^t a^{iD}dB^{iD}_u+\int _0^t a^i dB^{i}_u+\int _0^t a^{i\perp } dB_u^{i\perp }\nonumber \\&-\frac{1}{2}\int _0^t((a^{iD})^2+(a^i)^2+(a^{i\perp })^2) du\Big ) , \end{aligned}$$

where $B=(B^{iD},B^{i},B^{i\perp })$ is defined by (A.6), and

$$\begin{aligned} a^{iD}=-r{\bar{\alpha }}(\varepsilon _D^\star \sigma _D+\varepsilon _\pi ^\star o_M^\star \sigma _D^{-1}),\quad a^i=-r{\bar{\alpha }} \varepsilon _\pi ^\star o_M^\star w_i^\star \sigma _i,\quad a^{i\perp }=-r{\bar{\alpha }}\varepsilon _\pi o_M^\star w_{ i\perp }^\star , \end{aligned}$$

where $w^\star _{ i\perp }$ is the $w_{i\perp }$ computed with $w_i^*$, $1\le i\le n$. and the remaining parameters are in Definition 1.1 (iii).

#### Lemma A.10

For any $1\le i\le n$, the process $({\mathcal {N}}^i_t)_{t\ge 0}$ defined in (A.11) is a normalized stochastic discount factor for the *i*-th agent.

#### Proof

(A.9) is obviously satisfied for any ${\mathcal {H}}^i={\mathcal {N}}^i$, $1\le i\le n$. To prove the rest, we first show that the process

$$\begin{aligned} {\mathcal {M}}_t^i:={\mathcal {N}}_t^i P_t+\int _0^t{\mathcal {N}}_u^i D_u du \end{aligned}$$

is a local martingale. As ${\mathcal {M}}_t^i$ is an Itô integral, it suffices to show that its drift vanishes almost surely. By the product rule,

$$\begin{aligned} d{\mathcal {M}}_t^i=-r e^{-rt}{\mathcal {E}}_t^i P_t dt+{\mathcal {N}}_t^i dP_t+e^{-rt}P_t d{\mathcal {E}}_t^i+e^{-rt} d[P,{\mathcal {E}}^i]_t+e^{-rt} {\mathcal {E}}_t^i D_t dt, \end{aligned}$$

and the drift must be zero because ${\mathcal {E}}_t^i$ is a martingale. Therefore, the problem reduces to showing that the drift in

A.12 $$\begin{aligned} dZ_t^i:=dP_t-rP_tdt+D_tdt +\frac{d[P,{\mathcal {E}}^i]_t}{{\mathcal {E}}^i_t} \end{aligned}$$

vanishes. By the functional form of the price (1.8), the dynamics of the dividend and the consensus estimate in the system (A.8), and the fact that ${\hat{\pi }}_t^i$ and $\pi _t^w$ are indistinguishable (see Lemma A.8), it follows that the drift terms of $dZ_t^i$ are those of

$$\begin{aligned}&d(C+\varepsilon _D^\star D_t+\varepsilon _\pi ^\star d\pi _t^w)-r(C^\star +\varepsilon _D^\star D_t+\varepsilon _\pi ^\star \pi _t^w)dt+D_tdt\\ {}&+d[\varepsilon _D^\star \sigma _D dB^{iD}+\varepsilon _\pi ^\star o_M^\star (\sigma _D^{-1}dB^{iD}+w_i^\star \sigma ^{-1}dB^i+w_{i\perp }^\star dB^{i\perp }),a^{iD}dB^{iD}\\&+a^i dB^i+a^{i\perp }dB^{i\perp }]_t \end{aligned}$$

These terms are in turn given by the affine function

$$\begin{aligned}&\pi _t^w(\varepsilon _D^\star -\varepsilon _\pi ^\star (a+r))+D_t(1-\varepsilon _D^\star (k+r))\\ {}&\quad +\varepsilon _\pi ^\star a{\overline{\pi }}-rC^\star -r{\overline{\alpha }}\left[ (\varepsilon _D^\star \sigma _D+\varepsilon _\pi ^\star o_M^\star \sigma _D^{-1})^2+(\varepsilon _\pi ^\star o_M^\star )^2\sum _i \sigma _i^{-2}\right] . \end{aligned}$$

(Using the fact that $w_i^\star =\sigma _i^{-2}$ for $1\le i\le n$ and that the filter is stationary, that is $o_M(t)\equiv o_M^\star ={\overline{\sigma }}_\pi ^2$, see Lemma (iii).) The first two linear terms vanish by the definition of $\varepsilon _\pi ^\star$ and $\varepsilon _D^\star$. A straightforward but lengthy algebraic manipulation, featuring the explicit formula *C* in (1.16), yields that the third term also vanishes. Thus, the drift of (A.12) indeed vanishes and ${\mathcal {M}}_t^i$ is a local martingale.

Similar computations yield that the Brownian terms of $d{\mathcal {M}}_t$ are of the form

$$\begin{aligned} d{\mathcal {M}}_t^i={\mathcal {N}}_t^i \left( C_0 dB_t^{iD}+\sum _{j=1}^n C_j dB_t^j+P_t(D_0 dB_t^{iD}+\sum _{j=1}^n D_j dB_t^j)\right) , \end{aligned}$$

with some real constants $C_i,\,D_i$, $0\le i\le n$. As ${\mathcal {N}}_t^i$ is a geometric Brownian motion (discounted by $\exp (-rt)$), and $P_t$ is square-integrable (as sum of Ornstein-Uhlenbeck processes), the Cauchy-Schwarz inequality implies that ${\mathbb {E}}[[{\mathcal {M}}^i,{\mathcal {M}}^i]_t]<\infty$, for all $t\ge 0$. That is, ${\mathcal {M}}$ is a square-integrable ${\mathcal {F}}_t^i$-martingale, whence the martingale property (A.10) holds. $\square$

#### Lemma A.11

Let $(\theta _t,c_t)$ be an admissible strategy for the *i*-th agent. Then

A.13 $$\begin{aligned} {\mathcal {N}}_t^i X_t^i+\int _0^t{\mathcal {N}}^i_s c_s ds \end{aligned}$$

is an ${\mathcal {L}}^2$-martingale, and

A.14 $$\begin{aligned} \liminf _{T\rightarrow \infty }{\mathbb {E}}[{\mathcal {N}}^i_T X_T^i\mid {\mathcal {F}}_t^i]\ge 0. \end{aligned}$$

Consequently,

A.15 $$\begin{aligned} \limsup _{T\rightarrow \infty }{\mathbb {E}}\left[ \int _t^T {\mathcal {N}}_s^i c_s^ids\mid {\mathcal {F}}_t^i\right] \le {\mathcal {N}}_t^i X_t^i. \end{aligned}$$

#### Proof

First, we show the decomposition

A.16 $$\begin{aligned}&{\mathcal {N}}_t^i X_t^i+\int _0^t {\mathcal {N}}_s^i c_s^ids=x_0^i+\int _0^t \theta _sd\left( {\mathcal {N}}_s^i P_s+\int _0^s {\mathcal {N}}_u^iD_udu\right) \\\nonumber&\qquad +\int _0^t e^{-rs}(X_s-\theta _sP_s)d(e^{rs} {\mathcal {N}}_s^i). \end{aligned}$$

As ${\mathcal {E}}^i_t:=e^{rt}{\mathcal {N}}_t^i$ is by construction a square integrable martingale, the third term on the right side is a local martingale. The second term on the right side of (A.16) is also a local martingale, and so is ${\mathcal {N}}_t^i$ by the definition of the stochastic discount factor (Lemma A.10). Furthermore, as ${\mathbb {E}} [\int _0^t\theta _s^2 ds]<\infty$ and ${\mathcal {N}}_t^i P_t+\int _0^t {\mathcal {N}}^i_s D_s ds$ is an ${\mathcal {L}}^2$-martingale, by construction also (A.13) is an ${\mathcal {L}}^2$-martingale.

To prove (A.16), note that by the product formula and the self-financing property (1.11),

$$\begin{aligned} d({\mathcal {N}}_t^i X_t^i)+ {\mathcal {N}}_t^ic_t dt&=-r e^{-rt} {\mathcal {E}}_t^i X_t^idt+e^{-rt} X_t^id{\mathcal {E}}_t^i+e^{-rt} {\mathcal {E}}_t dX_t^i+ e^{-rt} d[{\mathcal {E}}^i,X^i]_t+c_t{\mathcal {N}}_t^i dt\\ {}&=e^{-rt} X_t^i d{\mathcal {E}}_t^i+ e^{-rt} \theta _t d[{\mathcal {E}}^i, P]_t+ e^{-rt}{\mathcal {E}}_t^i (\theta _t D_t dt-r \theta _tP_tdt+\theta _t dP_t). \end{aligned}$$

Hence, integration by parts yields

$$\begin{aligned} d({\mathcal {N}}_t^i X_t^i)+ {\mathcal {N}}_t^ic_tdt = e^{-rt}\theta _t d(P_t{\mathcal {E}}_t^i) + {\mathcal {E}}_t^i D_t dt - r\theta _t P_t e^{-rt} {\mathcal {E}}_t^i dt+e^{-rt}(X_t^i-\theta _t P_t)d{\mathcal {E}}_t^i . \end{aligned}$$

Once again, integration by parts allows to combine the first two terms on the right side to obtain (A.16), proving the first part of the statement.

Next, by the Cauchy-Schwarz inequality,

$$\begin{aligned} -{\mathbb {E}}[{\mathcal {N}}^i_T X_T^i\mid {\mathcal {F}}_t^i]&\le {\mathbb {E}}[\vert {\mathcal {N}}^i_T X_T^i\vert \mid {\mathcal {F}}_t^i ]\le \sqrt{{\mathbb {E}}[\vert {\mathcal {N}}^i_T\vert ^2\mid {\mathcal {F}}_t^i ]}\sqrt{{\mathbb {E}}[\vert X_T^i\vert ^2\mid {\mathcal {F}}_t^i ]}\\&={\mathcal {N}}_t^i e^{-rT+\frac{(T-t)}{2}\left( \vert a^{iD}\vert ^2+\vert a^i\vert ^2+\vert a^{i\perp }\vert ^2\right) }\sqrt{{\mathbb {E}}[\vert X_T^i\vert ^2 \mid {\mathcal {F}}_t^i ]}. \end{aligned}$$

Therefore property (A.14) follows from the admissibility condition (1.12).

The third part of the statement (A.15) follows from (A.13)-(A.14). $\square$

#### Proposition A.12

(Candidate strategy) Let $y^{i\star }=e^{-r \alpha _i x_0^{i}+\delta _0^i}$, where $\delta _0^i$ is defined in (1.19), and define a self-financing strategy with wealth $X^{i\star }_t$, $t\ge 0$, as

A.17 $$\begin{aligned} c_t^{i\star }=rX_t^{i\star }-\frac{\delta _0^i}{\alpha _i},\quad \theta ^{i\star }_t=\frac{{\bar{\alpha }}}{ \alpha _i },\quad t\ge 0, \end{aligned}$$

where $\delta _0^i$ is defined in (1.19). Then:

(i) (Admissibility) for each $1\le i\le n$, $(c_t^{i\star },\theta _t^{i\star })_{t\ge 0}$ is an admissible strategy and its wealth process $(X^{i\star }_t)_{t\ge 0}$ is
  A.18 $$\begin{aligned} X^{i\star }_t&= x_0^{i} +\left( \left( \varepsilon _\pi ^\star a{\bar{\pi }}-rC^\star \right) \frac{{\bar{\alpha }}}{\alpha _i}-c_0^i\right) t\nonumber \\&+\frac{{\bar{\alpha }}}{\alpha _i}\left( (\varepsilon _D^\star \sigma _D+\varepsilon _\pi ^\star o_M^\star \sigma _D^{-1}) B^{iD}_t+o_M^\star \varepsilon _\pi ^\star \sum _{j=1}^n \sigma _j^{-1}B_t^j\right) , \end{aligned}$$
  where the Brownian motion $B=(B^{iD},B^{i}, 1\le i\le n)$ is defined in (A.6) and
  $$\begin{aligned} c_0^i:=\frac{\beta _i-r}{r\alpha _i}+\frac{r{\bar{\alpha }}^2}{2\alpha _i} \left( (\varepsilon _D^\star )^2\sigma _D^2+2\varepsilon _D^\star \varepsilon _M^\star o_M^\star +(o_M^\star \varepsilon _\pi ^\star )^2 \nu ^\star \right) . \end{aligned}$$
(ii) The utility of the strategy is
  $$\begin{aligned} E\left[ \int _0^{\infty }e^{-\beta _i u}U(c_u^{i\star })du\Big |{\mathcal {F}}^i_0\right] =-\frac{y^{i\star }}{r \alpha _i}. \end{aligned}$$

#### Proof

To prove (i), note that the self-financing condition (1.11), Definition 1.1 (ii) and (iii), equation (1.16) and Lemma (iii) A.7 imply identity (A.18) for the wealth $X^{i\star }$. Using the definition of ${\mathcal {N}}_t^i$, which is a geometric Brownian motion, and of portfolio wealth, a Brownian motion with constant drift, it follows that $\lim _{T \rightarrow \infty }\frac{\log E[\vert X_T^{i\star }\vert ^2|{\mathcal {F}}^i_t]}{T}=0$, that is strictly smaller than the right hand side of (1.12), which is strictly positive by condition (1.15). The utility in (ii) is obtained by a straightforward computation. $\square$

#### Theorem A.13

(Duality) For any $1\le i\le n$, let $({\mathcal {N}}_t^i)_{t\ge 0}$ be the stochastic discount factor (A.11). Then

(i) The utility of the *i*-th agent satisfies
  $$\begin{aligned} E\left[ \int _0^{\infty }e^{-\beta _i u}U(c_u)du\Big |{\mathcal {F}}^i_0\right] \le -\frac{y^{i\star }}{r\alpha _i}= \frac{-e^{-r\alpha _ix_0^i+\delta _0^i}}{r\alpha _i}, \end{aligned}$$
  for any admissible strategy $(c_t,\theta _t)_{t\ge 0}$, where $\delta _0^i$ is defined in (1.19), and therefore the strategy in (A.17) is optimal for any $1\le i\le n$.
(ii) For any $1\le i\le n$, the optimal strategy (A.17) satisfies the transversality condition^8^,
  A.19 $$\begin{aligned} \lim _{T \rightarrow \infty }E\left[ \int _t^{T} {\mathcal {N}}_u^i {\widetilde{c}}_u^i du\Big |{\mathcal {F}}^i_t\right] = {\mathcal {N}}^i_t {\widetilde{X}}_t^i. \end{aligned}$$

#### Proof

Recall that for any $x\in {\mathbb {R}}$ and for any $y>0$,

$$\begin{aligned} U^i(x)\le {\widetilde{U}}^i(y)+x y, \end{aligned}$$

where $U^i(\cdot )=-e^{-\alpha _i \cdot }/\alpha _i$ is the utility function of the *i*-th agent and ${\widetilde{U}}^i(\cdot )$ is its Fenchel conjugate ${\widetilde{U}}^i (y)=\frac{y}{\alpha _i}(\log y -1)$ (cf. [7, Tables 4.1 and 4.2]). For any $0\le s \le t$ and $y>0$, the properties of the conjugate ${\widetilde{U}}$ yield

A.20 $$\begin{aligned} {\mathbb {E}}\left[ \int _s^T e^{-\beta _i u}U(c_u)\mid {\mathcal {F}}_s^i\right]\le & {} {\mathbb {E}}\left[ \int _s^Te^{-\beta _i u} {\widetilde{U}}(y e^{\beta _i u}{\mathcal {N}}^i_u)du\mid {\mathcal {F}}_s^i\right] \nonumber \\&+y{\mathbb {E}}\left[ \int _s^T {\mathcal {N}}^i_u c_u du\mid {\mathcal {F}}_s^i\right] \end{aligned}$$

and

$$\begin{aligned} E\left[ \int _s^t e^{-\beta _i u} {\widetilde{U}}(y e^{\beta _i u}{\mathcal {N}}^i_u)du\Big |{\mathcal {F}}^i_s\right]&=\frac{y}{\alpha _i}\Big \{\left( \log y -1\right) E\left[ \int _s^t {\mathcal {N}}^i_u du\Big |{\mathcal {F}}^i_s\right] + \\&\quad + \beta _i E\left[ \int _s^t u {\mathcal {N}}^i_udu \Big |{\mathcal {F}}^i_s\right] \\&\quad +E\left[ \int _s^t {\mathcal {N}}^i_u \log {\mathcal {N}}^i_u du\Big |{\mathcal {F}}^i_s\right] \Big \}. \end{aligned}$$

Set ${\mathcal {E}}_t^i:=e^{rt}{\mathcal {N}}_t^i$. By the conditional version of Fubini’s Theorem [3, page 13, Theorem 1.1.8],

$$\begin{aligned}&E\left[ \int _s^t e^{-\beta _i u} {\widetilde{U}}(y e^{\beta _i u}{\mathcal {N}}^i_u)du\Big |{\mathcal {F}}^i_s\right] \\&\quad =\frac{y}{\alpha _i}\Big \{\left( \log y -1\right) {\mathcal {E}}^i_s\int _s^t e^{-ru} du + + (\beta _i-r) {\mathcal {E}}^i_s\int _s^t u e^{-r u} du\\&\qquad +\int _s^t e^{-ru}E\left[ {\mathcal {E}}^i_u \log {\mathcal {E}}^i_u\Big |{\mathcal {F}}^i_s\right] du\Big \}. \end{aligned}$$

Setting $s=0$ and letting $t\rightarrow \infty$, it follows that^9^

A.21 $$\begin{aligned}&\lim _{t\rightarrow \infty }E\left[ \int _0^t e^{-\beta _i u} {\widetilde{U}}(y e^{\beta _i u}{\mathcal {N}}^i_u)du\Big |{\mathcal {F}}^i_0\right] \nonumber \\&\quad =\frac{y}{r\alpha _i}(\log (y)-1)+\frac{y}{r^2\alpha _i} (\beta _i-r)+\frac{y}{\alpha _i}\frac{\vert a^{iD}\vert ^2+\vert a^i\vert ^2+\vert a^{i\perp \vert ^2}}{2r^2}. \end{aligned}$$

The previous estimated employ limits for $T\rightarrow \infty$. Instead, the last term in (A.20) does not necessarily have a limit as $T\rightarrow \infty$, but the latter equation yields an estimate the utility of any admissible strategy, as follows. Using (A.15),

$$\begin{aligned} {\mathbb {E}}\left[ \int _0^\infty e^{-\beta _i u}U(c_u)\mid {\mathcal {F}}_0^i\right]&\le {\mathbb {E}}\left[ \int _0^\infty e^{-\beta _i u}{\widetilde{U}}(ye^{\beta _i u}{\mathcal {N}}^i_u)du\mid {\mathcal {F}}_0^i\right] \\&\quad +\limsup _{T\rightarrow \infty }y{\mathbb {E}}\left[ \int _0^T {\mathcal {N}}^i_u c_u du\mid {\mathcal {F}}_0^i\right] \\&\le {\mathbb {E}}\left[ \int _0^\infty e^{-\beta _i u} {\widetilde{U}}(y e^{\beta _i u}{\mathcal {N}}^i_u)du\mid {\mathcal {F}}_0^i\right] +x_0^{i}y. \end{aligned}$$

Thus, setting $y=y^{i\star }=e^{-r \alpha _i x_0^{i}+\delta _0^i}$ and using (A.21),

$$\begin{aligned}&\lim _{t\rightarrow \infty }E\left[ \int _0^t e^{-\beta _i u} {\widetilde{U}}(y^{i\star } e^{\beta _i u}{\mathcal {N}}^i_u)du\Big |{\mathcal {F}}^i_s\right] +x_0^i y^{i\star }\\&\quad =x_0^i y^{i\star }+\frac{y}{r\alpha _i}(\log (y)-1)+\frac{y}{r^2\alpha _i} (\beta _i-r)+\frac{y}{\alpha _i}\frac{\vert a^{iD}\vert ^2+\vert a^i\vert ^2+\vert a^{i\perp \vert ^2}}{2r^2}\\&\quad =\frac{e^{-r\alpha _i x_0^i+\delta _0^i}}{-r\alpha _i}=-\frac{y^{i\star }}{r\alpha _i}. \end{aligned}$$

Thus, the duality bound (A.20) implies that the utility of any strategy is bounded above by $-\frac{y^{i\star }}{r\alpha _i}$. As this utility is attained by the strategy in (A.17), the latter is indeed optimal, and part (i) follows.

Proof of part (ii): For any admissible strategy, the process in (A.13) is a martingale. As the optimal strategy (A.17) is admissible, this martingale property implies

A.22 $$\begin{aligned} {\mathbb {E}}\left[ \int _t^T {\mathcal {N}}_s^i c_s^{i\star }ids\mid {\mathcal {F}}_t^i\right] ={\mathcal {N}}_t^i X_t^{i\star }-{\mathbb {E}}[{\mathcal {N}}_T^i X_T^{i\star }\mid {\mathcal {F}}_t^i]. \end{aligned}$$

Due to (A.22), the claim (A.19) is equivalent to

A.23 $$\begin{aligned} \lim _{T\rightarrow \infty }{\mathbb {E}}[{\mathcal {N}}_T^i X_T^{i\star }\mid {\mathcal {F}}_t^i]=0. \end{aligned}$$

Using the vector-valued standard Brownian motion *B*, Proposition A.12 (that is, the wealth identity (A.18)) implies that, for some $c,d\in {\mathbb {R}}$ and $\kappa \in {\mathbb {R}}^{n+1}$,

$$\begin{aligned} X_T^{i\star }=c+dT+\kappa ^\top B_T . \end{aligned}$$

Moreover, for some $\lambda \in {\mathbb {R}}^{n+1}$, $({\mathcal {N}}^{i}_t)_{t\ge 0}$ equals

$$\begin{aligned} {\mathcal {N}}^i_T=e^{-rT} e^{\lambda B_T-\frac{1}{2}\Vert \lambda \Vert T} . \end{aligned}$$

(For simplicity, we suppress the dependence of all these constants on the *i*-th agent.) The martingale property of the stochastic exponential implies that

A.24 $$\begin{aligned} \lim _{T\rightarrow \infty }{\mathbb {E}}[(c+dT){\mathcal {N}}_T^i\mid {\mathcal {F}}_t^i]=\lim _{T\rightarrow \infty } e^{-r(T-t)} (c+dT) {\mathcal {N}}_t^i=0. \end{aligned}$$

Furthermore, by Itô’s formula applied to $B_T e^{\lambda ^\top B_T-\frac{1}{2}\Vert \lambda \Vert T}$ and the additivity of the martingale property,

$$\begin{aligned}&{\mathbb {E}}[\kappa ^\top B_T e^{-rT}e^{\lambda ^\top B_T-\frac{1}{2}\Vert \lambda \Vert T}\mid {\mathcal {F}}_t^i]\\&\quad =e^{-rT}\left( \kappa ^\top B_t e^{\lambda ^\top B_t-\frac{1}{2}\Vert \lambda \Vert t}+\lambda ^\top \kappa {\mathbb {E}}[\int _t^Te^{\lambda ^\top B_u-\frac{1}{2}\Vert \lambda \Vert u} du \mid {\mathcal {F}}_t^i]\right) \\&\quad =e^{-rT}\left( \kappa ^\top B_t+\kappa ^\top \lambda (T-t)\right) , \end{aligned}$$

whence

$$\begin{aligned} \lim _{T\rightarrow \infty }\kappa ^\top {\mathbb {E}}[ B_T e^{-rT}e^{\lambda ^\top B_T-\frac{1}{2}\Vert \lambda \Vert T}\mid {\mathcal {F}}_t^i]=0. \end{aligned}$$

In combination with the limit (A.24), the claim (A.23) follows, and so does (A.19). $\square$

The results established in Sect. 1 provide a complete proof of the main theorem, as follows:

#### Proof of Theorem 1.5

Proof of (i) – (ii): Let the price of the risky asset $P=(P_t)_{t\ge 0}$ be as in (i). Then each of the agents’ consumption-investment policies stated in (ii) is admissible (Proposition A.12 (i)) and optimal (Proposition A.18 (ii) and Theorem A.13 (i)). As the market clearing condition (1.13) is satisfied, the existence of an equilibrium in the sense of Definition 1.4 follows.

Proof of (iii): The value function is obtained similarly to the utility in the duality Theorem A.13 (i), using the arguments in its proof following (A.20). Part (iv) holds by applying the filtering Lemma A.7 (iii) with the values of the weights $w_i=\sigma _i^{-2}$ for $1\le i\le n$, (cf. the simplified equation (A.5)). $\square$

## Footnotes

1 For further developments on rational expectations equilibria, see [2, 4, 6, 8, 9, 12, 17, 21, 24].

2 Indeed, as *t* increases, $(D_t,\pi _t)$ converges in law to the bivariate Gaussian distribution with mean $({\bar{\pi }}/k, {\bar{\pi }})$ and covariance matrix

1.3 $$\begin{aligned} \begin{pmatrix} \frac{\sigma _D^2}{2k}+\frac{\sigma _\pi ^2}{2ak(a+k)} &{} \frac{\sigma _\pi ^2}{2a(a+k)}\\ \frac{\sigma _\pi ^2}{2a(a+k)} &{} \frac{\sigma _\pi ^2}{2a} \end{pmatrix} , \end{aligned}$$

hence the long-term correlation of $\pi _t$ and $D_t$ is positive.

3 The assumption that $\pi _0\sim {\mathcal {N}}({\overline{\pi }},{\overline{\sigma }}_\pi ^2)$ is not arbitrary, as ${\overline{\sigma }}_\pi ^2$ is chosen in equilibrium. (In particular ${\overline{\sigma }}_\pi ^2\ne \sigma _\pi ^2/2a$, the long-run volatility of the unobserved state of the economy, $\pi _t$). The particular choice of ${\overline{\sigma }}_\pi ^2$ made below implies stationary filters for the individual agent’s estimates, as well as for the market consensus, see Lemma (iii) A.7 and A.7.

4 That is, $\pi ^w$ is positive-recurrent with stationary distribution $\pi _\infty \sim {\mathcal {N}}({\bar{\pi }}, {\hat{\sigma }}^2_\pi /2a)$.

5 In Kyle’s continuous-time model, the insider’s information is eventually revealed at the final horizon, but remains partially concealed throughout the period.

6 We are indebted to an anonymous referee for suggesting this comparative-statics analysis.
